# Geographical accessibility to upper secondary education: an Italian regional case study

**DOI:** 10.1007/s00168-022-01146-6

**Published:** 2022-05-28

**Authors:** Giuseppe Bruno, Manuel Cavola, Antonio Diglio, Carmela Piccolo

**Affiliations:** Dipartimento di Ingegneria Industriale (DII), Università degli Studi di Napoli Federico II, Napoli, Italy

**Keywords:** I24 Education and Inequality

## Abstract

In this paper, a spatial analysis is performed to measure students’ access to the upper secondary education system. Based on the definition of quantitative indicators, the adopted approach is applied to an Italian regional case by exploiting the capabilities of a GIS software and using census tracts’ level data. The obtained results highlight geographical patterns of inequalities in access among students and shed light on the least served areas. Further analysis shows that accessibility reflects the degree of urbanization within the study region and that geographical distances are actual barriers to rural students since they are not compensated for by either economic status or the availability of digital infrastructures. The study offers empirical grounds to inform the decision-making process toward equity-in-access oriented interventions. Longer-term actions, as the activation of new schools (network expansion), the activation of new programs (service expansion) or the redistribution of their supply among the current network (network reorganization), as well as mid-term ones, like offering economic support for students’ mobility, or reinforcing digital connectivity, emerge as relevant to mitigate social exclusion.

## Introduction

In the last decades, governments and institutions across various western countries have been engaging in deep public spending reviews as part of broader austerity measures introduced to reduce the deficit and public debt (Alonso et al. 2015). From the organizational point of view, this has led to the adoption of a new paradigm for public administration, generally referred to as “New Public Management” (NPM, see Hood 1991, 1995), that put great emphasis on cost minimization and efficiency objectives. In this context, public authorities have implemented reforms to rationalize services in multiple sectors (e.g., education, healthcare, justice), with a general trend toward service capacity downsizing, facility closure, merging, and the consolidation of service provision in high densely populated areas. In light of this evidence, the NPM paradigm has been criticized for neglecting important public values, such as equity (Reiter and Klenk 2019), thus producing disparities in access to essential services among population groups. The recent COVID-19 pandemic has reinforced this criticism, stimulating a profound reflection on the territorial organization of public services. Notably, the very recent EU multiannual recovery plan allocates a large amount of the total budget for cohesion, resilience, and values “to strengthen economic, social and territorial cohesion,” focusing on disadvantaged regions and areas (European Commission 2021). Therefore, the debate around the accessibility to the Services of General Economic Interest—SGEI (COM 2011), is regaining momentum.

This work focuses on the education sector, which has been characterized by numerous structural interventions in the last decades. Following a so-called *sufficientarian approach*, the latter fostered a significant closure of schools or their consolidation in the most densely populated areas (Bruno et al. 2016), thus often exacerbating social differentiation (Teese et al. 2007a; b). In contrast to an *egalitarian approach*, which asserts the necessity of an equal distribution of resources or opportunities, *sufficientarianism* assumes a minimal level to be guaranteed and that inequalities beyond that point are acceptable. While sufficientarianism is undoubtedly justifiable in some cases, egalitarianism appears more suitable for evaluating accessibility to educational opportunities. Indeed, education services have an intrinsic positional value; this means that the increase of individual opportunities for someone determines the reduction of opportunities for others. Hence, disparities in access to educational services may potentially affect the acquisition of higher-level competencies and job positions, producing a potential socioeconomic divide.

As the provision of education services has recently represented one of the primary concerns of governments (Armitage and Nellums 2020; Azevedo et al. 2020), it is interesting to analyze the effects of such measures on the students’ spatial accessibility to schools. To this end, the availability of appropriate analysis tools to detect disparities is beneficial, as policymakers can exploit them to promote mitigation initiatives.

In this work, we focus on an Italian regional case study (Campania Region) to analyze geographical accessibility to the upper secondary education (USE) system. Based on the existing supply of schools and demographical data at the census tracts’ level, we propose a set of indicators that provide insights to understand how geographical disparities may constrain school choice and determine inequalities.

The remainder of the paper is organized as follows. In Sect. 2, the literature background and the contributions of the work are illustrated. Section 3 presents the methodological framework used, and Sect. 4 describes the case study. Then, results from the spatial analysis are presented in Sect. 5, while, in Sect. 6, the relationship between spatial and socioeconomic indicators is explored. Section 7 finally closes the paper with some concluding remarks and directions for further research.

## Literature background

Introduced in the health care sector by Penchasky and Thomas (1981), access is a complex concept involving five different dimensions: *availability* (adequacy of the system supply), *accessibility* (the relationship between the locations of providers and users), *accommodation* (how providers’ resources are organized to satisfy users’ demand), *affordability* (users’ perception of the costs of the offered services), and *acceptability* (users’ reaction to personal and practice providers’ features). In the field of education, this definition has been extended to include *adaptability*, i.e., the providers’ availability to adapt to individual ability (Tomaševski 2006), and *horizontality,* i.e., characteristic of prestige and quality across the system (McCowan 2016).

The above factors can be distinguished into two main categories, *spatial* and *aspatial*, depending on their geographical connotation. The former, which are the focus of the present work, include availability and accessibility. In general, the education literature mainly focused on aspatial ones; possibly, this is due to the predominant influence that, some decades ago, such dimensions had in influencing school choice. However, more recently, the re-organization of school networks has encouraged a growing academic interest in spatial inequality or injustice (Soja 2010; Eacott and Freeborn 2019; Tieken and Auldridge-Reveles 2019). As a result, many studies explicitly focused on geographical aspects to analyze socioeconomic phenomena or assess the effects of already implemented reforms. Various streams can be identified within this research area.

The most relevant one comprises works investigating the role of geography in influencing schools’ choices and determining disparity conditions. Harris et al. (2007) demonstrated, with a case study in Birmingham (UK), how pupils from different neighborhoods are more or less likely to attend their nearest public secondary school and how this attitude varies with the ethnic composition of the neighborhood itself. To this end, the authors adopted a logit model employing distances to the closest school as an accessibility indicator. Similarly, Yoshida et al. (2009), using data from the Adachi ward (Japan), examined how introducing the school choice program in junior high schools in Japan caused student sorting and disparities. Besides distances, an availability-based indicator was also used in that reference, namely the number of schools reachable within a given radius. Burgess et al. (2011) used proximity measures (i.e., distances to the nearest school) to investigate the case of England, arguing that the significant differences in the range of schools available to different families continue to be a significant barrier to reducing inequality of access. Chumacero et al. (2011) proposed an econometric framework to evaluate the determinants of school choice in the case study of Santiago (Chile). Using distances to the nearest school and the number of schools available within a given radius as access indicators, the authors demonstrated that distances and schools’ quality are (in trade-off) the most relevant factors. Hamnett and Butler (2011), employing students’ distances to secondary schools in East London (UK), proved the crucial role played by the place of residence in influencing school choice. Also, in a later work (Hamnett and Butler 2013), the same authors further highlighted the role distances play in the reproduction, intensification, or reduction of educational inequality. Distances were also the only geographical factor used by Singleton et al. (2011) to estimate schools’ catchment areas based on the home locations of pupils attending public primary school in England. Andersson et al. (2012), Thelin and Niedomysl (2015), and Lind (2019) analyzed the effects of the Swedish parliamentary reform to address secondary school choice, aimed at increasing available opportunities to students and encouraging competition within the education sector. In particular, Andersson et al. (2012) showed that the observed increase in distance to schools traveled by 15-years-olds does not reflect increased school choice, which—on the contrary—depends on individual or neighborhood-level social and ethnic factors. Thelin and Niedomysl (2015) provided an empirical investigation underlining how geographical factors, namely distances, schools’ locations (central or outer city areas), and availability of public means of transport, constitute a significant share of the overall relative importance in school choice decision making. According to the authors, this result would highlight significant flaws in the assumptions that motivated the reform. Finally, Lind (2019), focusing on districts of northern Sweden, highlighted that increased competition generated higher costs per capita and fewer available programs in smaller municipalities, thus reducing the corresponding number of students completing their studies compared with larger cities. For the analysis, the author employed only geographical data related to schools’ and pupils’ locations (i.e., small vs. large municipalities).

Notably, other works investigated the relationship between geographical aspects and students’ performance. Engberg et al. (2012) showed that the adverse effects on students’ performance determined by schools’ closure due to a consolidation process in an unnamed US district could be reduced by closing low-performing schools and transferring students to higher-performing ones. Falch et al. (2013) analyzed the relationship between graduation from upper secondary education and travel times to the nearest school in Norway. In particular, they underlined a negative (but weak) effect of travel time on students’ performance.

A final research stream involves descriptive studies of students' spatial accessibility to compulsory education. Williams and Wang (2014) implemented a two-step floating catchment area method (hence, using travel distances and the number of schools available in a given radius) to calculate an accessibility score for each school of Baton Rouge (Mississippi, USA). They detected variations in disparities in the single city areas with different socioeconomic conditions. Lee and Lubienski (2017) exploited the same method and indicators to examine the impact of school closures on the socio-spatial distribution of equitable access to primary schools following the school closure policy pursued in Chicago. Curl et al. (2015) analyzed differences between objective and subjective measures of journey time accessibility to a range of local destinations in the UK, including primary and secondary schools, highlighting how objective journey times are shorter than subjective ones. Gao et al. (2016) employed travel distances to describe the inequality of compulsory education from the perspective of imbalanced spatial distribution in China, using shortest travel distances. Hernandez (2018) explored the unequal access to education facilities (primary, secondary, vocational) among different social classes in Montevideo (Uruguay), evaluating the number of opportunities using public transport and the travel time by public transport to the closest education facility. Kelobonye et al. (2019) employed an “accessible-opportunities” approach to examine the relative accessibility, and the spatial equity of five key urban land uses in Perth (Australia), including primary and secondary education. In practice, they evaluated the number of schools available to students from their locations within given travel times. Results proved that accessibility to education facilities presents striking differences, considering travel times using private cars or public transport.

From the analysis of the extant contributions, we note remarkable attention to the issue of disparity in access to secondary education opportunities and a consensus about the role of barriers played by geographical distances (or travel times). However, these results generally come from limited case studies, mainly focused on urban areas. Also, the number of indicators employed appears somewhat limited, being restricted to single availability or accessibility measures. For this reason, we propose studying a detailed regional case to analyze disparities in access to the multi-vocational secondary school system, taking into account (and stratifying our analysis by) the full range of available educational programs. We also consider a variety of indicators to capture the multi-dimensional aspects of the problem to different geographical extents (at the municipal and sub-municipal levels) and, consequently, the complexity of policies to be implemented to achieve higher territorial cohesion.

Hence, in our opinion, the main contributions of the present work to the literature are threefold: (1) the use and calculation of numerous spatial indicators which enable to understand the multifaceted characteristics to consider in the evaluation of inequalities in educational access; (2) the detailed articulation of the case study, based on a very fine discretization of the study area and highly detailed geographic information, which allows an adequate representation of the phenomenon under investigation; (3) the application in the context of the USE system, characterized by schools providing different programs, in which accessibility has to be evaluated for both single programs and the whole system.

## An approach to analyzing accessibility to the upper secondary education system

Upper secondary education (USE) is the third level of education, according to the International Standard Classification of Education (ISCED—level 3, UNESCO 2011). It aims at preparing students to access tertiary education or provide skills relevant to entering the labor market. At this level, programs are more differentiated, and offer students an increased range of options and streams. Specifically, each upper secondary school (USS) may provide one or more upper secondary programs (USPs). Hence, a school can be seen as a *multi-service facility*, and the school network may be represented as a *multi-service facility network*. Based on this premise, the spatial accessibility to the USE system can be analyzed considering single programs/services (USPs) or the whole school network. To this end, we introduce two groups of indicators:

- *Service Accessibility Indicators*, assessing the diffusion over a given region of schools (USSs) providing specific programs (UPSs) and their proximity to students;
- *Network Accessibility Indicators*, assessing the accessibility of students to the whole USS network and, hence, to the whole gamma of provided services (USPs).

To define the proposed indicators, it is fundamental to effectively represent the spatial distribution of students and the supply of educational programs. Specifically, we need to consider the set of discrete nodes where students are located (i.e., residential addresses or aggregate demand points), the current locations of USSs (geographical coordinates), the USPs offered by each school, and the distances between each demand node and each school. In order to compare accessibility indicators at different geographical extents, we refer to the standard classification introduced by Eurostat for the statistical subdivision of European countries (Nomenclature of Units for Territorial Statistics-NUTS). In particular, we define metrics concerning the more refined partition in Local Authorities Units (LAU), usually corresponding to single municipalities, in such a way that they can be then aggregated at provincial (NUTS 3) and regional (NUTS 2) levels.

At this aim, we use the following notation:

- $I$: set of demand nodes where students are located, indexed by $i$;
- $J$: set of nodes where USSs (*facilities*) are located, indexed by $j$;
- $K$: set of USPs offered by all USSs, indexed by $k$;
- $a_{jk}$: binary label indicating whether an USP $k \in K$ is available at USS $j \in J$;
- $p_{i}$: potential students living in node $i \in I$;
- $d_{ij}$: distance between the demand node $i \in I$ and the USS $j \in J$;
- $M$: set of municipalities within the considered region, indexed by $m$;
- $I_{m}$: subset of demand nodes $i \in I$ located within the boundaries of a given municipality $m \in M$;
- $J_{m}$: subset of USSs $j \in J$ located within the boundaries of a given municipality $m \in M$.

### Service accessibility indicators

The first group of indicators detects the *availability* of single programs in the study area, highlighting population groups served within their living municipality.

- $n_{mk}$: *presence index*—this indicator measures whether a municipality $m \in M$ hosts at least one school providing the USP $k \in K$ ($n_{mk} = \min \left( {1;\mathop \sum \nolimits_{{j \in J_{m} }} a_{jk} } \right)$. Hence, it can assume only values equal to 1 or zero.
- $N_{k}$: *served areas*—number of municipalities hosting at least a school providing the program $k \in K$ within their boundaries ($N_{k} = \mathop \sum \nolimits_{m \in M} n_{mk}$). Such areas are considered *fully served* with reference to service $k \in K$.
- $P_{k}$: *served population*—students having at least a USS offering the USP $k \in K$ within the boundaries of their living municipality ($P_{k} = \mathop \sum \nolimits_{{m \in M:n_{mk} = 1}} \mathop \sum \nolimits_{{i \in I_{m} }} p_{i}$). Such students are considered *fully served* with reference to the service $k \in K$.

The second group of indicators measures the *proximity* of students to schools offering given programs. Specifically, we measure the accessibility of students in node $i \in I$ to program $k \in K$ as the distance from the closest school $j \in J$ offering $k$ itself $\left( {d_{i}^{k} = \mathop {\min }\nolimits_{{j \in J:a_{jk = 1} }} d_{ij} } \right)$. Based on single accessibility conditions, we define the following indicators:

- $d_{m}^{k}$: *service accessibility distance—*it expresses the distance that students living in municipality $m \in M$ are expected to travel, on average, to reach the closest USS $j \in J$ providing USP $k \in K$ $\left( {d_{m}^{k} = \frac{{\mathop \sum \nolimits_{{i \in I_{m} }} p_{i} d_{i}^{k} }}{{\mathop \sum \nolimits_{{i \in I_{m} }} p_{i} }}} \right)$;
- $\alpha^{k} \left( d \right)$: *service accessibility function*—the percentage of students in the study region having the closest USS offering USP $k$ within distance $d$ ($\alpha^{k} \left( d \right) \to \left[ {0,1} \right]$). The function provides the distribution of the population by their accessibility conditions, similarly to the Lorenz curve used for income distribution.

### Network accessibility indicators

This group of indicators provides information on the accessibility of students to the whole school network, by aggregating the information referred to the single programs. Specifically, we define:

- $n_{i}^{ } \left( r \right)$: *available opportunities—*number of alternative USPs $k \in K$ accessible within a given distance $r$ to students living in node $i \in I$ $\left( {n_{i}^{ } \left( r \right) = \left| {\left\{ {k \in K:d_{i}^{k} \le r} \right\}} \right|} \right)$. In order to compute this indicator at the municipal level, we consider the number of schools that, on average, a student living in municipality $m \in M$ has within distance $r$ $\left( {n_{m}^{ } \left( r \right) = \frac{{\mathop \sum \nolimits_{{i \in I_{m} }} p_{i} n_{i}^{ } \left( r \right)}}{{\mathop \sum \nolimits_{{i \in I_{m} }} p_{i} }}} \right)$. While $n_{i}^{ } \left( r \right)$ assumes discrete values between 0 and $\left| K \right|,$ $n_{m}^{ } \left( r \right)$ assumes real values in the same range.
- $R_{i}^{ }$: *full-service radius*—distance within which students living in $i \in I$ reach the full set of opportunities, in terms of USPs $\left( {R_{i} = \mathop {\max }\nolimits_{k \in K} d_{i}^{k} } \right)$. At the municipal level, the radii characterizing the demand nodes within a given municipality $m \in M$ are weighted by the corresponding student population $\left( {R_{m}^{ } = \frac{{\mathop \sum \nolimits_{{i \in I_{m} }} p_{i} R_{i}^{ } }}{{\mathop \sum \nolimits_{{i \in I_{m} }} p_{i} }}} \right)$.
- $\overline{d}_{i}$: *expected accessibility*—distance that students living in $i \in I$ are expected to travel to attend their selected USP. Given the probability $w_{ik}$ that students in $i \in I$ select the USP $k \in K$, it can be expressed as the weighted average of the single accessibility distances $d_{i}^{k}$ $\left( {\overline{d}_{i} = \mathop \sum \nolimits_{k \in K} w_{i}^{k} d_{i}^{k} } \right)$. At the municipal level, such indicator is computed by weighting the expected accessibilities of the single demand nodes by the corresponding student population $\left( {\overline{d}_{m} = \frac{{\mathop \sum \nolimits_{{i \in I_{m} }} p_{i} \overline{d}_{i} }}{{\mathop \sum \nolimits_{{i \in I_{m} }} p_{i} }}} \right)$.

Note that the above indicators can be applied to measure the accessibility to schools at any educational level. When applied to a lower level, i.e., where the supply is not differentiated and only one program is offered $(\left| K \right| = 1$), all the schools can be considered equivalent, and the network accessibility indicators are not needed.

## Description of the case study

We focus our analysis on the Italian context and, specifically, on regional education systems (NUTS2 level). This choice is motivated by the fact that the competence of secondary schools is delegated to regional authorities that can take decisions based on the peculiarities and the specific needs of the territories. Moreover, this does not affect the quality of the analysis as the mobility of students among regions is negligible for the upper secondary schools.

Following the reform introduced in 2010 at the national level (Gazzetta Ufficiale 15/06/2010, available at: https://www.gazzettaufficiale.it/), the Italian USE involves two macro-categories of schools: lyceums (*licei*) and technical and vocational institutes (*istituti tecnici e professionali*), that, respectively, offer general and vocational programs. Lyceums focus on core topics (such as Italian, Latin, history, and philosophy) and differ based on specific specializations. Precisely, it is possible to distinguish among: *classic lyceum*, dedicated to humanities; *scientific lyceum*, focused on scientific studies (i.e., mathematics, physics, chemistry, biology, and computer science); *linguistic lyceum*, that puts emphasis on modern foreign languages (i.e., English, French, Spanish, and German); *human sciences lyceum*, mainly devoted to relational, behavioral, and educational topics (i.e., pedagogy, anthropology, psychology, and sociology). On the other side, vocational institutes are more oriented toward basic skills to enter the labor market. In particular, *technical institutes* provide vocational education in the field of economics and technology, while *vocational institutes* in service, industry, and crafts sectors.

For our analysis, we focus on the Campania Region, which is the second most populated region in Italy, with about 6 million of inhabitants (density of 425 inhabitants per km^2^). To calculate the introduced indicators, we implement the following methodological steps (see Barbarisi et al. 2019; Bruno et al. 2020):

1. Delimitation of the study region;
2. Definition of the (multi-service) facility network;
3. Demand representation;
4. Distance matrix calculation.

### Delimitation of the study region

**Fig. 1:**
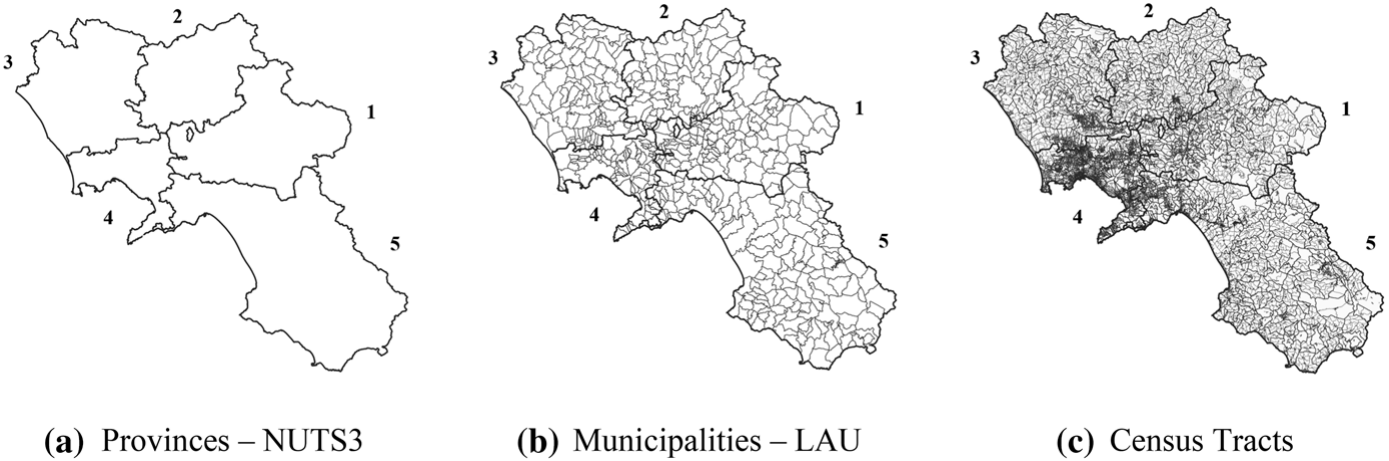
Subdivisions of the study region in statistical units

We consider the area included in the administrative boundaries of the Campania Region (NUTS 2), islands excluded (i.e., Capri, Ischia, and Procida). Indeed, the mobility of students toward schools positioned on the mainland is negligible due to the existence of significant geographical barriers. The study region is subdivided into the five provinces (NUTS 3) and the 541 municipalities (LAU) depicted in Fig. 1a, b. For simplicity, from now on, each province (Avellino, Benevento, Caserta, Napoli, and Salerno) will be labeled with a unique ID code (from 1 to 5).

### Definition of the (multi-service) facility network

**Fig. 2:**
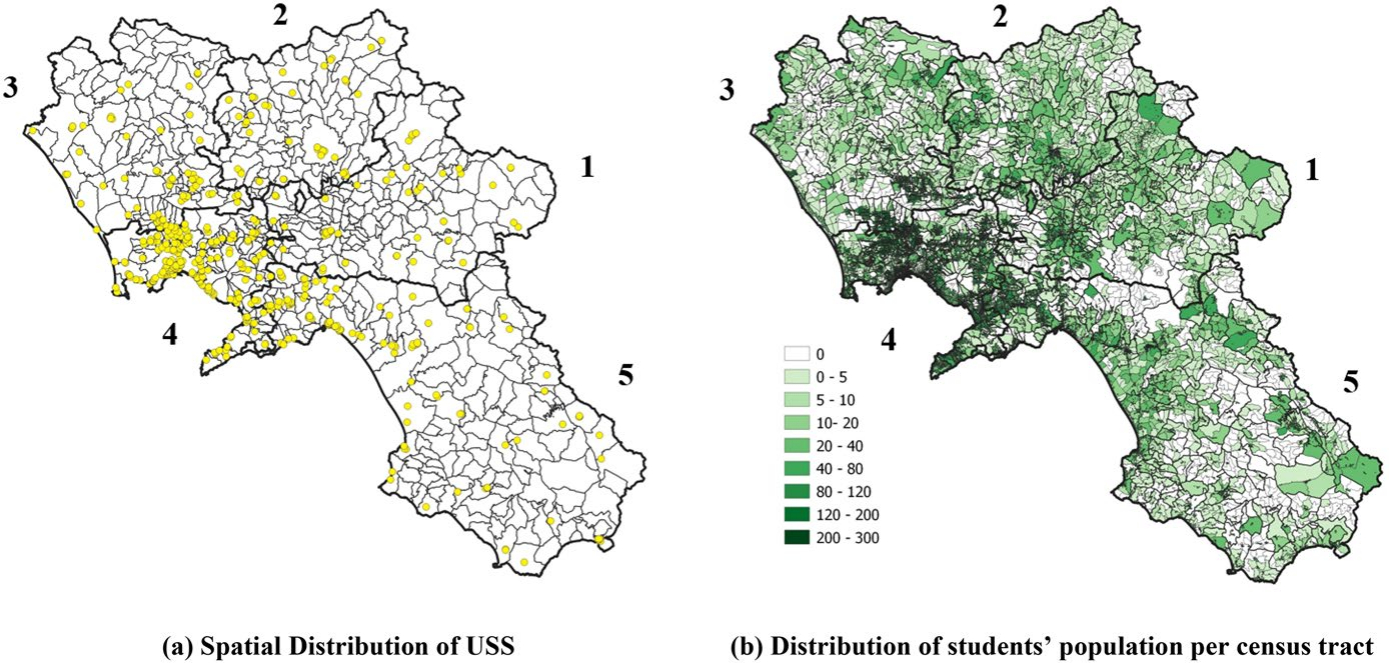
Demand and supply representation

We retrieved the school network data from the official database of the Italian Ministry of Education, University and Research (MIUR—Ministero dell’Università e della Ricerca, available at www.dati.istruzione.it), which provides the address and the offered USPs for each school. From the whole set of 918 USSs located in the study region, those devoted to students with particular needs and located at particular sites (i.e., hospitals or prisons) were excluded, thus considering a final set $J$ of 692 USSs. Their coordinates were obtained through customized requests to Google Maps performed using MATLAB. In Fig. 2a, the spatial distribution of the USSs (yellow dots) over the study region is shown. Table 1 reports the distribution of schools and programs per province. Considering that each school may offer multiple programs, the total number of USPs (917) is higher than the number of USSs (692). It is possible to notice that the overall number of lyceums and vocational programs are comparable (431 and 413, respectively) and that scientific ones are the most diffused among lyceums.

**Table 1:** Number of USS and USPs by province

ID	Province	USSs	USPs						Total
			Lyceums				Institutions		
			Classic (CL)	Scientific (SL)	Linguistic (LL)	Human science (HSL)	Vocational (VI)	Technical (TI)	
1	Avellino	72	10	20	6	8	21	29	104
2	Benevento	67	7	16	4	4	16	24	76
3	Caserta	96	14	28	12	13	23	25	131
4	Napoli	293	33	78	48	46	71	94	399
5	Salerno	164	18	33	19	14	57	53	207
	Total	692	82	175	89	85	225	188	917
			431				413		

### Demand representation

To effectively represent the nodes where potential students are located and minimize the errors related to the spatial accessibility measures, we refer to the most refined discretization of the national territory in census tracts provided by the National Institute of Statistics (ISTAT). Figure 1c shows the subdivision of the study region in its 18,849 census tracts. We associate each census tract with the living population in the age group 15–19 years (ISTAT 2011) and assume such population concentrated in its centroid. Figure 2b depicts the distribution of students per census tract. In Table 2, we report the demographic characteristics of the single provinces and some indicators that capture the density of supply in the study region.

**Table 2:** Demand data

ID	Province	Number of municipalities	Number of census tracts	Population	Population (15–19 years)	Extension (Km.^2^)	USSs	USSs per municipality	USSs per 1,000 students	USSs per 100 km.^2^
		(a)	(b)	(c)	(c')	(d)	(e)	(e)/(a)	[(e)/(c')] *1000	[(e)/(d)] *100
1	Avellino	118	1,684	424,707	24,142	2,806	72	0.61	2.98	2.57
2	Benevento	78	1,487	278,795	16,026	2,080	67	0.86	4.18	3.22
3	Caserta	104	2,311	900,229	57,088	2,651	96	0.92	1.68	3.62
4	Napoli	83	8,876	2,960,692	191,645	1,179	293	3.53	1.53	24.85
5	Salerno	158	4,491	1,081,806	62,969	4,954	164	1.04	2.60	3.31
	Total	541	18,849	5,646,229	351,870	13,670	692	1.28	12.98	37.57

### Distance matrix calculation

Distances ${d}_{ij}$ between the census tracts’ centroids ($i\in I$) and schools ($j\in J$) were determined as the shortest paths on the road network through customized requests to an open-source Geographic Information System (18,849 rows and 692 columns). For each program $k\in K$, the sub-matrix of distances between centroids and the sub-set of schools providing that service $\left({J}_{k}=\left\{j\in J:{a}_{jk=1}\right\}\right)$ was extracted to calculate service accessibility indicators.

## Analysis of the results

**Fig. 3:**
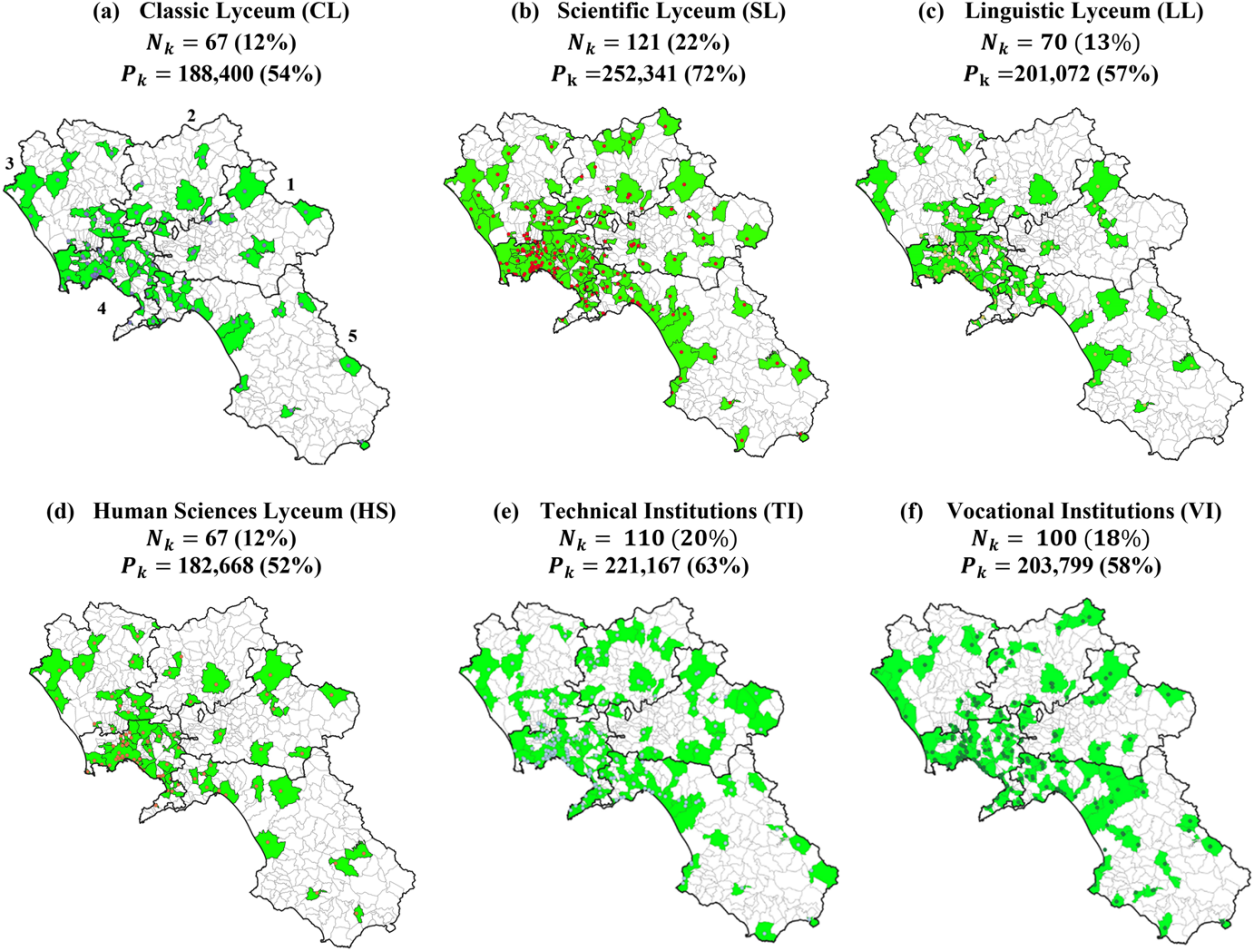
Number ($$N_{k}$$) and population ($$P_{k} )$$ of fully served municipalities by each USP

The analysis is performed through the calculation of the indicators introduced above. Figure 3 reports maps highlighting the municipalities fully served by each USP $k\in K$ (*presence index*, ${n}_{mk}$), with the indication of their total number (${N}_{k}$) and population (${P}_{k}$). It is possible to notice that the scientific lyceum is the most diffused, serving 72% of the regional population, while the less diffused are the classic and the human sciences lyceums, serving 54% and 52% of the potential users, respectively. Moreover, while most of the municipalities in the province of Naples (ID 4) are fully served, the supply of programs is less diffused in the other provinces.

**Fig. 4:**
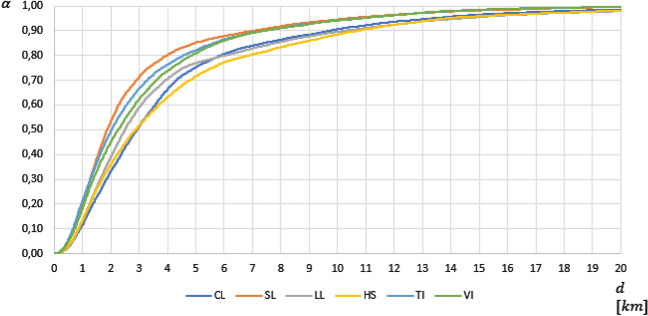
Service accessibility functions for each type of USP

Figure 4 depicts the *service accessibility functions* for each type of program $k\in K$. We observe that the accessibility function related to scientific lyceums (orange line) dominates the others, thus denoting the best accessibility conditions. They cover around 85% of the student population within 5 km, while the human sciences lyceums (HS) cover only 70% of students (yellow line) within the same distance. Moreover, it can be underlined that, for each program, most of the students are covered within 20 km ($\alpha$ is very close to 1.0). The picture does not display the residual portions of students positioned farther than this threshold distance. In order to give evidence of the worst-case conditions, Table 3 indicates the maximum distance per program, i.e., the 100th percentile of the corresponding distributions of the accessibility distances $\left({d}^{k}\left(\alpha =1.00\right)=\underset{i\in I}{\mathrm{max}}\left\{{d}_{i}^{k}\right\}\right)$. It emerges that the Linguistic and Human Science lyceums are characterized by very high maximum distances, equal to 51.6 km and 47.2 km, respectively. Instead, scientific, vocational, and technical programs have such values within 30 km.

**Table 3:** Average and maximum accessibility distances (in km) for each USP

	CL	SL	LL	HS	TI	VI
Avg	4.26	3.02	4.31	4.56	3.20	3.36
Max	34.43	29.38	51.56	47.22	28.19	28.19

**Fig. 5:**
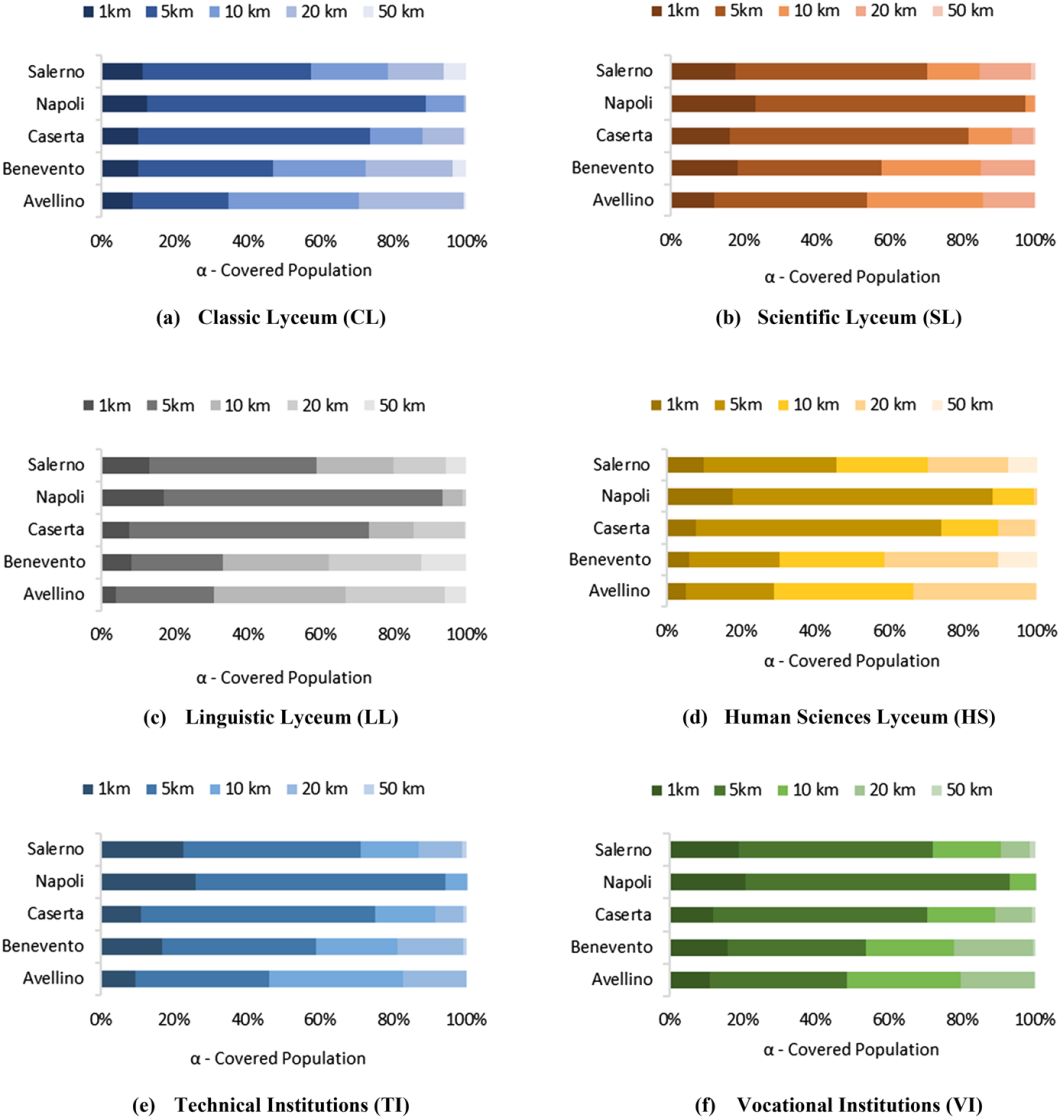
Accessibility functions for each type of USP and province

To better highlight disparities among territories, Fig. 5 displays the cumulative distributions of distances for each province and program. In the sub-figures, each bar is associated with a province and indicates the (cumulative) shares of students covered within given (increasing) distances. For each program, the darkest part of the bar represents the percentage of the population covered within 5 km. As expected, significant disparities emerge between the province of Naples (ID 4) and the cases of Avellino (ID 1) and Benevento (ID 2).

We now focus our attention on the so-called *network accessibility indicators* (see Sect. 3.2). These measures represent, in different ways, the probability that students access the complete set of alternatives and make a free choice about the program to attend.

**Fig. 6:**
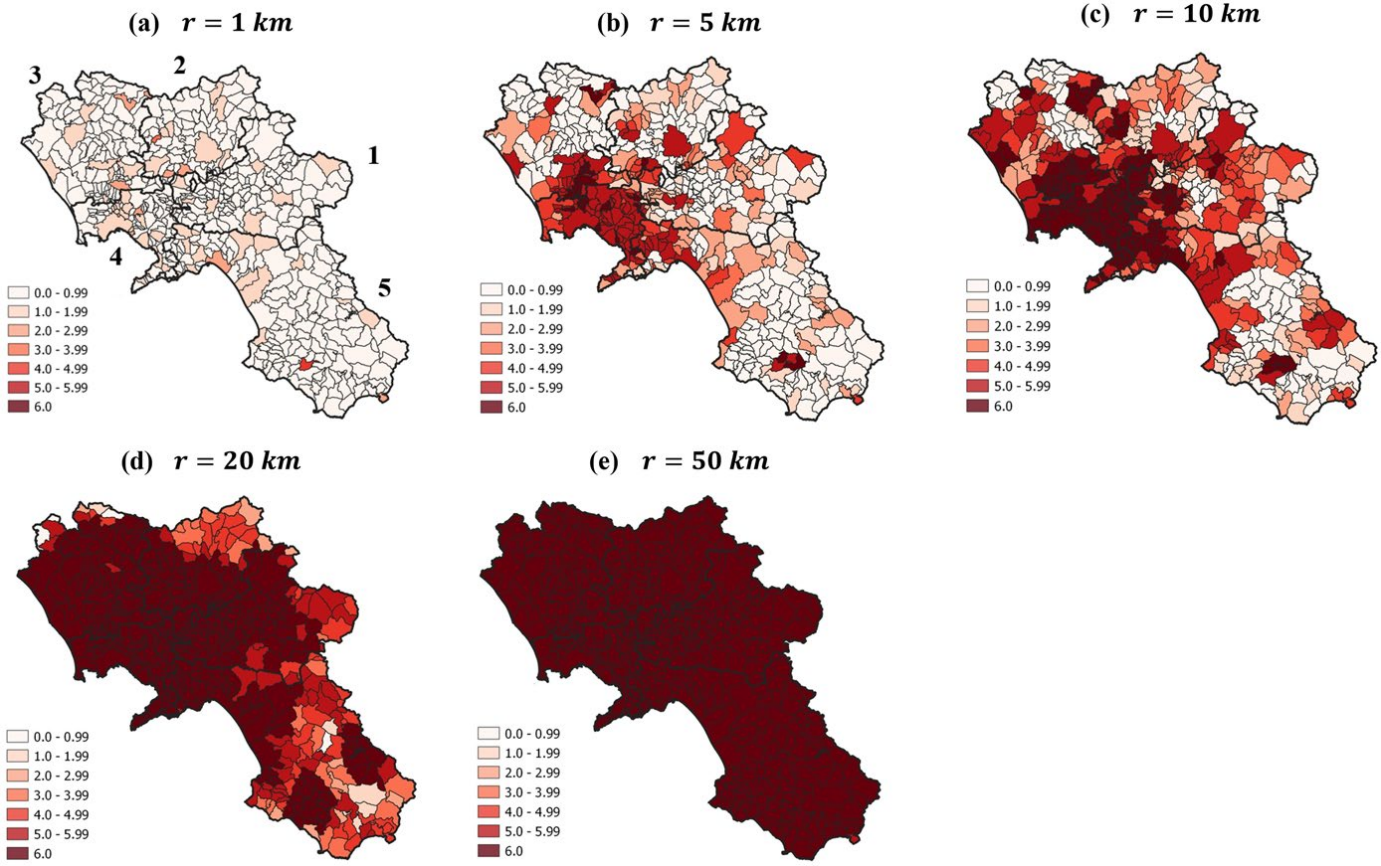
Number of different USPs available within the distance $r$ ($n_{m} \left( r \right))$

In Fig. 6, we report five maps highlighting the average number of opportunities (in terms of USPs) available for each municipality ($n_{m}^{ } \left( r \right)$) within different distances $r$ (1, 5, 10, 20, 50 km). The emerging differences are remarkable. Indeed, while most students in the province of Naples (ID 4) have access to the complete set of opportunities within 5 km, others have very limited options within the same distance, especially in the most peripheral areas. This produces a substantial exclusion from given educational programs for certain groups of students.

**Fig. 7:**
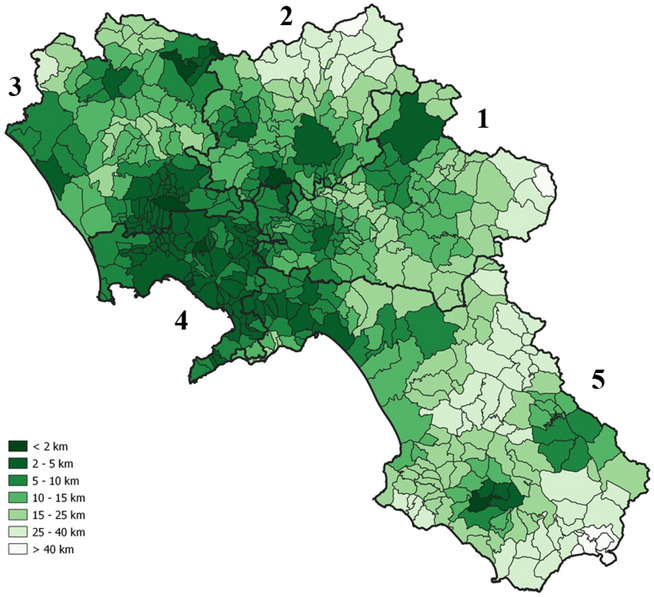
Full-service radius (in km)-distance to access the full gamma of educational program ($$R_{m} )$$

The full-service radius indicator ($R_{m}^{ }$), shown in Fig. 7, confirms this evidence. Indeed, in the province of Naples (ID 4) it is sufficient to travel at most 5 km to access all the USP, while in the others, the radius increases significantly. See, for example, the case of students living in the southern part of Salerno (ID 5), whose full-service radius is between 20 and 50 km.

**Fig. 8:**
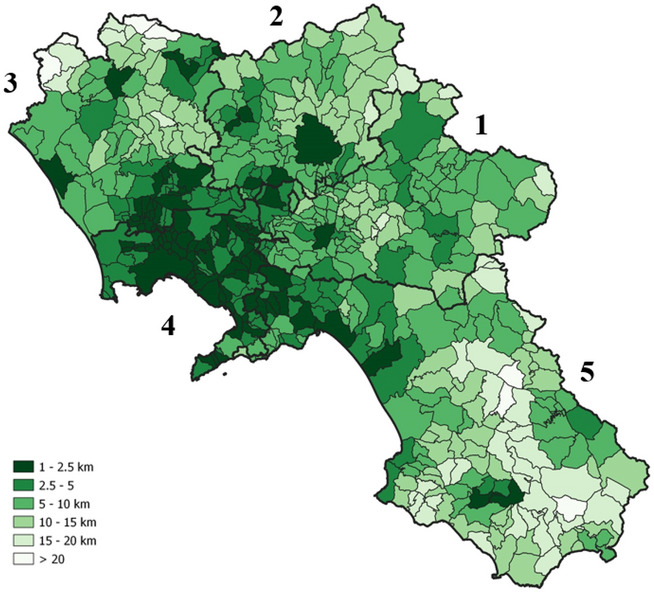
Expected travel distances $\overline{d}_{m}$ (in km) to access the selected USP

Considering that USPs have different levels of attractions and the distribution of students among them is not uniform, it may be interesting to analyze the indicator $\overline{d}_{m}$, in which the accessibility distances to single USPs are weighted by the corresponding probabilities to be selected by students. For the latter, we used the enrolment rates at the national level as a proxy. This way, we expect to smooth the local effects related to various constraints (e.g., admission policies, geographical barriers, to name a few)*.* In Fig. 8, the values of the indicator $\overline{d}_{m}$ are mapped. Of course, since the weighted distance privileges the most available USPs, we note again that, while in the province of Naples (ID 4), the expected distance from the chosen school is between 1 and 2.5 km, in the south of the Region it increases to 20 km and more. The “hotspots” in terms of accessibility in the two maps (Figs. 7 and 8) are almost coincident.

## Disparities in access to upper secondary education

This section aims to understand whether-and to what extent-inequalities in students’ access depend on the degree of urbanization within the study area. To this end, we adopt the classification provided by Eurostat (2011), which associates municipalities (and hence census tracts) with a unique code reflecting their population densities: one, for urban areas; two, for mid-densely populated areas; three, for rural areas. In practice, we replicated and stratified our analysis by considering this additional information. Next, we structure our results according to the different aspects we are focusing on.

### Degree of urbanization

Table 4 shows the number ($$N_{k} )$$ and the population ($$P_{k}$$) (both in percentages) of fully served municipalities by each USP $$k \in K$$ per degree of urbanization. Note that the total percentage values reported in rows 4 and 8 correspond to those shown in Fig. 3.

**Table 4:** Number ($$N_{k} )$$ and population ($$P_{k}$$) of served municipalities by each USP per degree of urbanization

Indicator	Degree of urbanization	CL (%)	SL (%)	LL (%)	HS (%)	TI (%)	VI (%)
$N_{k}$	1 (Urban)	4.99	9.43	6.47	6.28	6.65	5.73
	2	4.81	8.13	4.81	4.07	8.32	7.39
	3 (Rural)	2.59	4.81	1.66	2.03	5.36	5.36
	Total	12.38	22.37	12.94	12.38	20.33	18.48
$P_{k}$	1 (Urban)	41.50	54.29	45.57	42.82	46.34	42.39
	2	9.67	13.84	9.85	7.36	13.24	12.33
	3 (Rural)	2.37	3.58	1.72	1.74	3.27	3.20
	Total	53.54	71.71	57.14	51.91	62.85	57.92

**Fig. 9:**
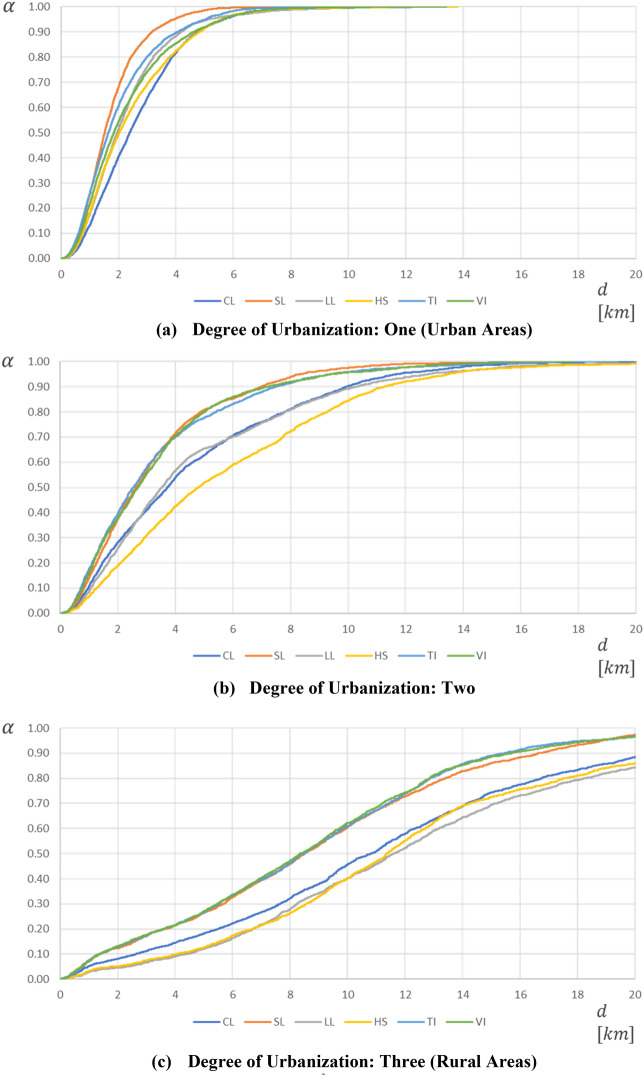
Accessibility functions ($\alpha^{k} \left( d \right))$ for each degree of urbanization and USP

The table highlights that most USSs are located in urban or mid-densely populated areas. Indeed, except for Technical and Vocational Institutions (columns 7 and 8), the percentage of fully served municipalities ($N_{k}$) in rural areas is almost half that in urban areas for each USP $k$. Besides, we note that rural areas account only for a small percentage of fully served population $(P_{k} ,$ row 7), which equals-at most-3.58% for Scientific Lyceums. In simple terms, the availability of educational programs is significantly higher in urban areas. This fact reflects very heterogeneous accessibility conditions, which are depicted, for each degree of urbanization and USP in Fig. 9. Table 5 reports the corresponding average and maximum accessibility distances.

**Table 5:** Average and maximum accessibility distances (in km) for each degree of urbanization and USP

	Degree of urbanization	CL	SL	LL	HS	TI	VI
Avg	1 (Urban)	2.67	1.75	2.31	2.47	2.02	2.29
	2	4.69	3.32	4.92	5.82	3.44	3.42
	3 (Rural)	11.43	8.88	13.21	12.52	8.68	8.67
Max	1 (Urban)	13.10	12.10	13.25	13.81	13.32	13.39
	2	20.78	19.03	51.56	30.30	19.35	17.81
	3 (Rural)	34.43	29.38	48.76	47.22	28.19	28.19

From Fig. 9, it is evident how accessibility worsens, for each USP, as the degree of urbanization increases. For instance, in the case of the Scientific Lyceums, 98% of urban students are covered within 5 km (Fig. 9a). However, this percentage reduces to 80% in mid-densely populated areas (Fig. 9b) and only to 26% in rural areas (Fig. 9c). The worst served students, living in rural areas, have the closest scientific lyceum at 29.38 km and must travel, on average, almost 9 km to access this program, namely 7 km more than urban students (Table 5). Similar evidence is found for the other USPs, thus proving that spatial inequalities in terms of geographical accessibility exist.

Looking at the overall gamma of educational programs, Tables 6 shows the calculation of the network accessibility indicators defined in Sect. 2. In particular, it reports the average number of programs available to students within a given sample of distances ($n_{i} \left( r \right)$), aggregated by degree of urbanization; the distances students should travel to reach all the educational programs ($R_{i}$), and the expected distance to attend the selected program ($\overline{d}_{i}$). For the two latter indicators, the minimum, maximum, and average values aggregated by degree of urbanization are indicated.

**Table 6:** Network accessibility indicators by degree of urbanization

		$$r \left( {{\text{km}}} \right)$$					
		1.00	2.50	5.00	10.00	20.00	50.00
$n_{i} \left( r \right)$	1 (Urban)	1.21	3.97	5.63	5.98	6.00	6.00
	2	0.73	2.37	4.19	5.53	5.98	6.00
	3 (Rural)	0.33	0.66	1.23	3.09	5.49	6.00

		Min	Max	Average
$R_{i}$	1 (Urban)	0.28	13.81	3.48
	2	0.29	51.56	7.09
	3 (Rural)	0.47	48.76	15.73

		Min	Max	Average
$\overline{d}_{i}$	1 (Urban)	0.18	11.93	2.01
	2	0.27	17.31	3.60
	3 (Rural)	0.28	26.85	9.07

Again, severe disparities can be noticed. Students in urban areas access, on average, the full gamma of educational programs within 3.48 km. Indeed, the number of different USPs available between 2.50 and 5.00 km increases from 3.97 to 5.63. In rural areas, instead, the average number of different USPs approximates the maximum (i.e., 6.00) only for distances higher than 20.00 km. Note, in fact, that students living in these zones should travel, on average, 15.73 km to reach all the USPs, that is, five times the distances traveled by urban residents.

### Per capita income

**Fig. 10:**
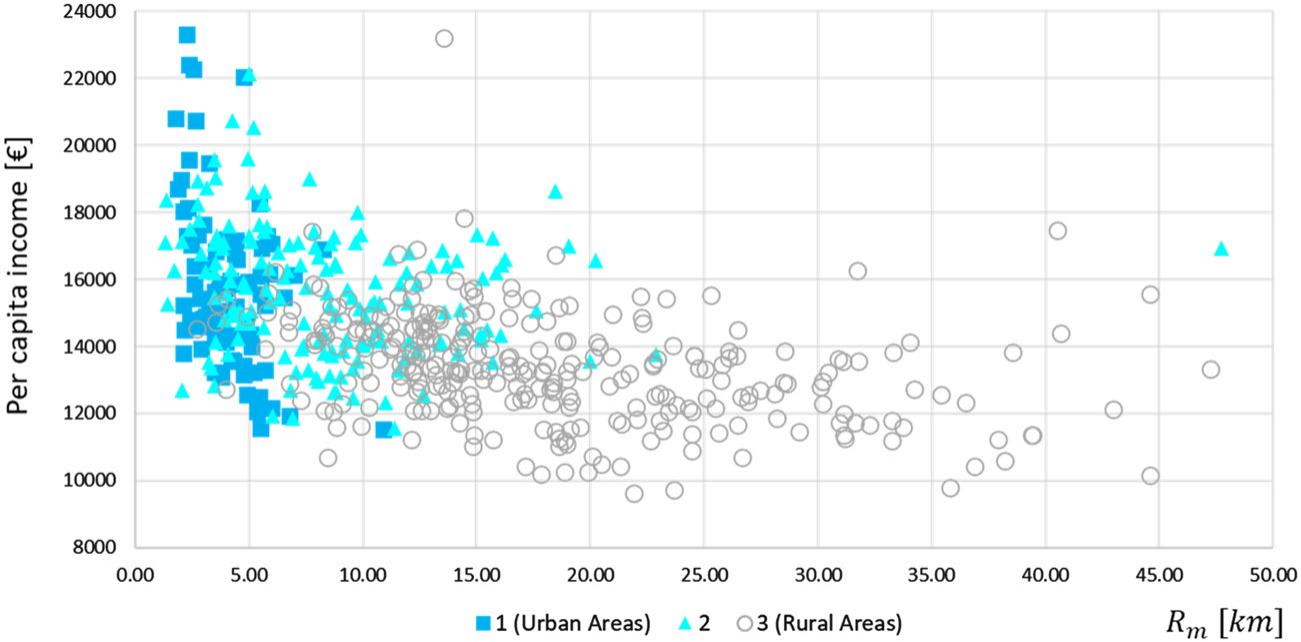
Per capita income versus students’ accessibility by degree of urbanization

Notably, differences in spatial access reflect students’ socioeconomic conditions. To support this claim, we considered the per capita income data by municipality provided by ISTAT (available at: http://dati.istat.it), and we detected the correlation with accessibility indicators. In Fig. 10, each municipality-thematized according to its degree of urbanization—is represented as a point whose coordinates are the related per capita income and the full-service radius $R_{m}$, i.e., the minimum distance students should cover to access the whole gamma of USPs.

In general, a negative correlation between income and accessibility emerges, as lower-income municipalities are typically associated with worse accessibility conditions (i.e., higher distances). Urban municipalities (blue squares) are characterized by higher per capita income and lower accessibility distances than rural ones (blank circles). In practice, while low-income students in urban areas still benefit from very good accessibility ($R_{m}$ slightly higher than 10 km in the worst case), this does not hold for their rural peers. As such, geographical barriers in access actually exist for rural students, which bear higher transportation costs regardless of their less affluent economic status.

We should also note that this correlation is not completely explained by the degree of urbanization (and, hence, population density). To shed light on this aspect, Table 7 reports, for each degree of urbanization, the average per capita income, calculated across municipalities characterized by distances $R_{m}$ comprised within some given ranges. The corresponding number of municipalities (which depends on $R_{m}$ only) is indicated in brackets. Accordingly, “N/A” is reported when no municipalities fall within a distance range.

**Table 7:** Average per capita income (in €) by degree of urbanization and distances to reach the full gamma of educational programs ($R_{m}$)

Degree of urbanization	$R_{m} \left[ {{\text{km}}} \right]$					
	$<$ 5.00	5.00–10.00	10.00–20.00	20.00–30.00	30.00–40.00	$\ge$ 40.00
1 (Urban)	16,173 (63)	14,813 (20)	11,525 (1)	N/A (0)	N/A (0)	N/A (0)
2	16,332 (43)	15,523 (69)	15,132 (47)	14,634 (3)	N/A (0)	16,944 (1)
3 (Rural)	14,592 (6)	14,159 (35)	13,618 (155)	12,732 (63)	12,302 (29)	13,842 (6)

The table shows that the average per capita income decreases as accessibility worsens within the same degree of urbanization (a few outliers are found for higher values of the indicator $R_{m}$). In practice, as the correlation persists after conditioning it to population densities, we can conclude that lower-income students suffer from worse accessibility conditions in both urban and rural areas.

For brevity, we do not report on the other calculated indicators, nor do we detail the correlations per each USP. However, similar considerations can be drawn.

### Availability of digital resources

**Fig. 11:**
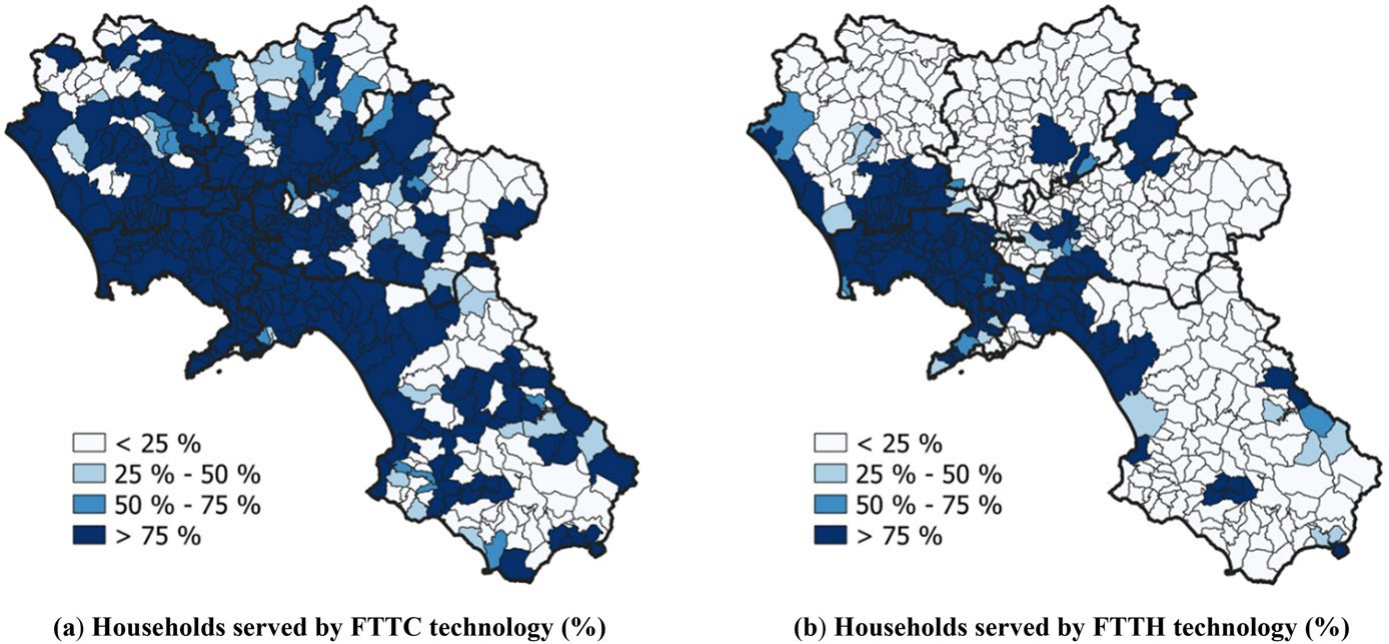
Broadband coverage indicators’ distributions by municipality

It is then interesting to analyze if geographical barriers are compensated, to some extent, by the availability of digital resources. The recent developments following the spread of the COVID-19 pandemic and the consequent lockdown have posed at the heart of the (inter)national debate the topic of distance learning and, hence, the availability of proper digital resources for students. In this view, we explored a “digital” dimension of accessibility, considering the data on *Broadband Coverage* provided by AGCOM (*Autorità per le Garanzie nelle Comunicazioni*), i.e., the regulator and competition authority for the communication industries in Italy. These data (available at: https://maps.agcom.it/) refer to the performance of existing internet network infrastructures and the availability of internet services to households at the municipal level. In particular, we focused on the *Connection typology* dataset, expressing the percentage of households served by FTTC (*Fiber-To-The-Cabinet*) and FTTH (*Fiber-To-The-Home*) technologies, whose distribution is displayed in Fig. 11.

In order to give evidence of the relationship between digital access and spatial accessibility, Table 8 reports, in the same fashion as Table 7, the average percentage of households served by FTTC or FTTH technology.

**Table 8:** Average percentage of households served by FTTC and FTTH technologies by degree of urbanization and distances to reach the full gamma of educational programs ($R_{m}$)

Technology	Degree of urbanization	$R_{m} \left[ {{\text{km}}} \right]$					
		$<$ 5.00	5.00–10.00	10.00–20.00	20.00–30.00	30.00–40.00	$\ge$ 40.00
FTTC	1 (Urban)	99.99% (63)	100.00% (20)	98.49% (1)	N/A (0)	N/A (0)	N/A (0)
	2	97.86% (43)	96.84% (69)	83.68% (47)	97.22% (3)	N/A (0)	100.00% (1)
	3 (Rural)	83.41% (6)	60.87% (35)	51.60% (155)	35.68% (63)	26.70% (29)	53.37% (6)
FTTH	1 (Urban)	99.32% (63)	79.87% (20)	28.07% (1)	N/A (0)	N/A (0)	N/A (0)
	2	80.70% (43)	44.60% (69)	23.22% (47)	0.00% (3)	N/A (0)	100.00% (1)
	3 (Rural)	46.95% (6)	9.41% (35)	6.01% (155)	1.75% (63)	0.00% (29)	13.52% (6)

As the table shows, the above percentages tend to decrease as distances increase, i.e., users in the worst accessibility conditions are also underserved in terms of connection technologies. Focusing on a single distance range, we notice that rural areas have poorer infrastructures (percentages decrease as urbanization degree increases). Note that, for values of the indicator $R_{m}$ higher than 10.00 km, the percentage of households served by FTTC connections in rural areas is often far below 50.00%. In particular, FTTC does not cover almost 75% of households within the 29 rural municipalities having a value of $R_{m}$ in the range 30.00–40.00 km. Also, such a condition applies to about 50% of households living in the six rural least-served municipalities in terms of spatial accessibility ($R_{m} \ge$ 40.00 km).

Although to a different extent, similar considerations can be drawn for the FTTH technology, which is less diffused—in general—in the study area (see Fig. 11b). FTTH does not serve any household living in rural municipalities characterized by a value of $R_{m}$ in the range 30.00–40.00 km. Very low percentages are found in the other cases: specifically, only 1.75% and 13.52% of households are served by FTTH for $R_{m} \in \left[ {20.00, 30.00} \right[$ km and $R_{m} \ge 40.00$ km, respectively. We also note that no households are served in three mid-densely populated municipalities for $R_{m} \in$ [20.00–30.00] km.

In practice, digital resources do not compensate for poorer spatial access and pose further (digital) barriers to students’ access to upper secondary education. Specifically, geographical distances are barriers to students living in rural areas since they are forced to travel more to access USPs despite a lower-income status and a significant digital divide. Although spatial access reflects the urban–rural aspect, we highlight that attention should also be given to mid-densely populated areas, where the percentage of households equipped with performing connections is low, and distance learning might not be viable.

## Discussion and concluding remarks

The analysis has highlighted how it is possible to detect “hotspots” characterized by a significant accessibility divide to upper secondary education. In the analyzed case, the worst accessibility conditions are associated with students living in less densely populated areas, generally suffering more severe economic difficulties and a higher digital divide due to underdeveloped digital infrastructure.

As underlined in the literature background, the described situation is not an exception. Indeed, in the last decades, the trend toward the closure and the consolidation of schools in high densely populated areas has led to a marginalization of peripheral regions. Even if such policies reduced public expenditure in the short-term horizon, they caused long-term adverse effects in terms of territorial cohesion. However, the recent pandemic has encouraged a different way of rethinking global land use with more rational and sustainable exploitation of territorial resources. According to the Next Generation EU (NGEU) program, national governments defined multi-annual plans to foster territorial cohesion and restore the economic development of disadvantaged areas. In this context, equal access to the compulsory education system would represent a fundamental pillar.

Our analysis showed the importance of a monitoring system based on the evaluation of quantitative indicators able to provide a comprehensive analysis of the phenomenon. In Italy, a specific public national agency has been founded for promoting economic development, cohesion, and social solidarity ((Territorial Cohesion Agency—https://www.agenziacoesione.gov.it/lagenzia/). It undertakes ad hoc actions to remove economic and social imbalances by enhancing cohesion policy planning, coordination, and monitoring. Moreover, our analysis may drive decision makers toward the definition of effective interventions to reduce the identified disparities.

As concerns the direct mitigation of the geographical barriers, our evidence can support the optimal location of new schools (*network expansion*) by identifying the sites that would improve students’ accessibility. Also, they can support the activation of new programs in appropriate locations (*service expansion*) or the redistribution of the supply among the existing schools (*network reorganization*). Of course, as such interventions have a long-lasting impact and require significant economic and human resources mobilization, they need to be sustainable. Building new schools in marginal areas with few students is not realistic. Such interventions should be integrated into broader strategic plans to restore the economic development of marginal areas and increase their attractiveness.

Besides, other measures may be adopted to mitigate geographical barriers in the shorter term. Usually, they consist of subsidized transportation fees for commuting students or subsidized rents (*economic support for student mobility*). These are conceived as horizontal instruments and may be ineffective if they do not consider the peculiarities of the areas where more disadvantaged students live. In this regard, the evidence of our analysis and the awareness of accessibility conditions in different areas could help design tailored support strategies. For instance, if the areas with the worst accessibility conditions are also not well served by transportation infrastructures, the decision maker should be aware that subsidies for transportation will likely fail.

Finally, as the diffusion of the COVID pandemic has raised the question of distance learning as a complementary teaching dimension, we also considered digital connectivity as a further dimension of access to the educational service (*digital connectivity exploitation*). Our analysis showed that, in general, worse geographical accessibility to the USE system is not compensated by digital resources. Usually, students in the worse accessibility conditions live in areas characterized by poor digital infrastructure. In this sense, our study may support policymakers in defining priority interventions in terms of investments in digital infrastructures.

Different directions for future research can be explored. Firstly, the analysis could be extended by considering additional access dimensions (i.e., availability, accommodation, acceptability, affordability, and adaptability). Moreover, other case studies may be explored to compare different contexts and policies. Finally, it would be interesting to analyze, within given territorial contexts, the geographical accessibility to an extended set of services of general interest, thus verifying the recurrence of “hotspots.” This would allow identifying critical areas exposed to risks of negative trends in a medium-long time horizon and defining priority interventions.

## Data Availability

Data can be made available by the authors upon request.

## References

[CR1] Alonso JM, Clifton J, Díaz-Fuentes D (2015). Did new public management matter? An empirical analysis of the outsourcing and decentralization effects on public sector size. Public Manag Rev.

[CR2] Andersson E, Malmberg B, Östh J (2012). Travel-to-school distances in Sweden 2000–2006: changing school geography with equality implications. J Transp Geogr.

[CR3] Armitage R, Nellums LB (2020). Considering inequalities in the school closure response to COVID-19. Lancet Glob Health.

[CR4] Azevedo JP, Hasan A, Goldenberg D, Ikbal SA, Geven K (2020) Simulating the potential impacts of COVID-19 school closures on schooling and learning outcomes: a set of global estimates. World Bank Report

[CR5] Barbarisi I, Bruno G, Diglio A, Elizalde J, Piccolo C (2019). A spatial analysis to evaluate the impact of deregulation policies in the pharmacy sector: evidence from the case of Navarre. Health Policy.

[CR7] Bruno G, Esposito E, Genovese A, Piccolo C (2016). Institutions and facility mergers in the Italian education system: models and case studies. Socioecon Plann Sci.

[CR8] Bruno G, Cavola M, Diglio A, Piccolo C (2020). Improving spatial accessibility to regional health systems through facility capacity management. Socio Econ Plan Sci.

[CR9] Burgess S, Greaves E, Vignolesc A, Wilsond D (2011). Parental choice of primary school in England: what types of school do different types of family really have available to them?. Policy Stud.

[CR10] Chumacero RA, Gómez D, Paredes RD (2011). I would walk 500 miles (if it paid): vouchers and school choice in Chile. Econ Educ Rev.

[CR11] COM (2011) A quality framework for services of general interest in Europe. Communication from the European Commission n.900, 20 Dec, 2011

[CR12] Curl A, Nelson JD, Anable J (2015). Same question, different answer: a comparison of GIS-based journey time accessibility with self-reported measures from the National Travel Survey in England. Comput Environ Urban Syst.

[CR13] Eacott S, Freeborn A (2019). Regional and rural school consolidation: a scoping study of research literature. Int J Educ Manag.

[CR14] Engberg J, Gill B, Zamarro G, Zimmer R (2012). Closing schools in a shrinking district: Do student outcomes depend on which schools are closed?. J Urban Econ.

[CR15] European Commission, The EU’s 2021–2027 long-term budget and NextGenerationEU: facts and figures, Publications Office, 2021. https://data.europa.eu/doi/10.2761/808559. Accessed on Dec 16, 2021

[CR16] Eurostat (2011) Degree of urbanization (DEGURBA). www.eur-lex.europa.eu. Accessed on Dec 16, 2021

[CR17] Falch T, Lujala P, Strøm B (2013). Geographical constraints and educational attainment. Reg Sci Urban Econ.

[CR18] Gao Y, He Q, Liu Y, Zhang L, Wang H, Cai E (2016). Imbalance in spatial accessibility to primary and secondary schools in china: guidance for education sustainability. Sustainability.

[CR19] Hamnett C, Butler T (2011). ‘Geography matters’: the role distance plays in reproducing educational inequality in East London. Trans Inst Br Geogr.

[CR20] Hamnett C, Butler T (2013). Distance, education and inequality. Comp Educ.

[CR21] Harris R, Johnston R, Burgess S (2007). Neighborhoods, ethnicity and school choice: developing a statistical framework for geodemographic analysis. Popul Res Policy Rev.

[CR22] Hernandez D (2018). Uneven mobilities, uneven opportunities: Social distribution of public transport accessibility to jobs and education in Montevideo. J Transp Geogr.

[CR23] Hood C (1991). Stabilization and cutbacks: a catastrophe for government growth theory?. J Theor Polit.

[CR24] Hood C (1995). Contemporary public management: a new global paradigm?. Public Policy Admin.

[CR25] ISTAT (2011) Dati del censimento generale della popolazione italiana (Italian national census data). www.istat.it Accessed on June 15, 2020

[CR26] Kelobonye K, McCarney G, Xia JC, Swapan MSH, Mao F, Zhou H (2019). Relative accessibility analysis for key land uses: a spatial equity perspective. J Transp Geogr.

[CR27] Lee J, Lubienski C (2017). The impact of school closures on equity of access in Chicago. Educ Urban Soc.

[CR28] Lind T (2019). Upper secondary schools and sparsity: the case of northern Sweden. Scand J Educ Res.

[CR30] McCowan T (2016). Three dimensions of equity of access to higher education. Compare J Compar Int Educ.

[CR31] Penchasky R, Thomas J (1981). The concept of access: definition and relationship to customer satisfaction. Med Care.

[CR32] Reiter R, Klenk T (2019). The manifold meanings of ‘post-New Public Management’—a systematic literature review. Int Rev Adm Sci.

[CR34] Singleton AD, Longley PA, Allen R, O’Brien O (2011). Estimating secondary school catchment areas and the spatial equity of access. Comput Environ Urban Syst.

[CR35] Soja EW (2010). Seeking spatial justice.

[CR36] Teese R, Lamb S, Duru-Bellat M (2007). International studies in educational inequality, theory and policy volume 1: educational inequality: persistence and change.

[CR37] Teese R, Lamb S, Duru-Bellat M (2007). International studies in educational inequality, theory and policy volume 2: inequality in education systems: persistence and change.

[CR38] Thelin M, Niedomysl T (2015). The (ir) relevance of geography for school choice: evidence from a Swedish choice experiment. Geoforum.

[CR39] Tieken MC, Auldridge-Reveles TR (2019). Rethinking the school closure research: school closure as spatial injustice. Rev Educ Res.

[CR40] Tomaševski K (2006). Human rights obligations in education: the 4-A scheme.

[CR41] UNESCO (2011) International Standard Classification of Education (ISCED). http://uis.unesco.org. Accessed on Dec 16, 2021

[CR42] Williams S, Wang F (2014). Disparities in accessibility of public high schools, in metropolitan Baton Rouge, Louisiana 1990–2010. Urban Geogr.

[CR43] Yoshida A, Kogure K, Ushijima K (2009). School choice and student sorting: Evidence from Adachi Ward in Japan. Jpn Econ Rev.

